# Effusion attenuates the effect of synovitis on radiographic progression in patients with hand osteoarthritis: a longitudinal magnetic resonance imaging study

**DOI:** 10.1007/s10067-020-05341-8

**Published:** 2020-08-29

**Authors:** W. Damman, R. Liu, M. Reijnierse, F. R. Rosendaal, J. L. Bloem, M. Kloppenburg

**Affiliations:** 1 Department of Rheumatology, Leiden University Medical Center, C1-R, PO Box 9600, 2300 RC Leiden, The Netherlands; 2 Radiology, Leiden University Medical Center (LUMC), Leiden, The Netherlands; 3 Clinical Epidemiology, Leiden University Medical Center (LUMC), Leiden, The Netherlands

**Keywords:** Effusion, Hand osteoarthritis, Magnetic resonance imaging, Synovitis

## Abstract

An exploratory study to determine the role of effusion, i.e., fluid in the joint, in pain, and radiographic progression in patients with hand osteoarthritis. Distal and proximal interphalangeal joints (87 patients, 82% women, mean age 59 years) were assessed for pain. T2-weighted and Gd-chelate contrast-enhanced T1-weighted magnetic resonance images were scored for enhanced synovial thickening (EST, i.e., synovitis), effusion (EST and T2-high signal intensity [hsi]) and bone marrow lesions (BMLs). Effusion was defined as follows: (1) T2-hsi > 0 and EST = 0; or 2) T2-hsi = EST but in different joint locations. Baseline and 2-year follow-up radiographs were scored following Kellgren-Lawrence, increase ≥ 1 defined progression. Associations between the presence of effusion and pain and radiographic progression, taking into account EST and BML presence, were explored on the joint level. Effusion was present in 17% (120/691) of joints, with (63/120) and without (57/120) EST. Effusion on itself was not associated with pain or progression. The association with pain and progression, taking in account other known risk factors, was stronger in the absence of effusion (OR [95% CI] 1.7 [1.0–2.9] and 3.2 [1.7–5.8]) than in its presence (1.6 [0.8–3.0] and 1.3 [0.5–3.1]). Effusion can be assessed on MR images and seems not to be associated with pain or radiographic progression but attenuates the association between synovitis and progression.

**Table:** 

**Key Points** *• Effusion is present apart from synovitis in interphalangeal joints in patients with hand OA.* *• Effusion in finger joints can be assessed as a separate feature on MR images.* *• Effusion seems to be of importance for its attenuating effect on the association between synovitis and radiographic progression.*

**Key Points**

*• Effusion is present apart from synovitis in interphalangeal joints in patients with hand OA.*

*• Effusion in finger joints can be assessed as a separate feature on MR images.*

*• Effusion seems to be of importance for its attenuating effect on the association between synovitis and radiographic progression.*

## Introduction

Where OA was formerly considered a purely mechanical disorder, nowadays the consensus is that in OA also low-grade inflammation is implicated [1]. Imaging studies with ultrasound and magnetic resonance (MR) have shown that synovitis and bone marrow lesions (BMLs) play a role in the pathogenesis of hand osteoarthritis (OA), since these inflammatory features are associated with pain and radiographic progression [2–6]. However, their precise role remains unclear since treatments with anti-inflammatory drugs such as biologicals and corticosteroids have largely been unsuccessful. Whether effusion, another inflammatory feature which is often seen with synovitis, is implicated, is unclear.

What causes joint effusion, i.e., excess fluid in the joint, is not understood. Osmotic pressure in the tissue versus the joint cavity, decreased drainage, and pressure of surrounding tissue seem of importance [7, 8]. Joint effusion is often seen with inflammatory reactions such as in erosive (inflammatory) hand OA [9, 10] but also in OA joints without warmth or tenderness or in clinically healthy joints [11]. These suggest different triggers for effusion, such as mechanics or inflammation.

A MR study that compared inflammatory features in finger joints between healthy volunteers and patients with rheumatoid or psoriatic arthritis suggested that joint effusion most likely reflects pathology [12]. In hand OA, the presence and role of effusion are rarely studied. Few studies scored effusion, likely since it is not included in the scoring methods as a separate feature [13, 14]. A study in finger OA that used both ultrasound and MR and an ultrasound study in hand OA suggested that assessment of effusion as a separate feature is valid and reliable and that effusion occurs frequently [2, 3, 15].

The aim of our exploratory study was to study the role of MRI-defined joint effusion in patients with hand OA. We defined effusion using 1.5-tesla MR images from our secondary care cohort and we studied the association of effusion, and effusion in combination with synovitis, with joint pain and radiographic progression. We hypothesized that the presence of effusion reflects inflammation and therefore reinforces the known association between synovitis and joint pain and progression.

## Materials and methods

### Study population

We included patients from our center diagnosed by their treating rheumatologists with primary hand OA in the HOSTAS (Hand OSTeoArthritis in Secondary care) study [16]. The present analysis concerned patients, included March 2011 to October 2012, who received a contrast-enhanced MRI at baseline and of whom 2-year follow-up radiographs, were available [6]. Written informed consent was obtained from all participants. The study was approved by the Leiden University Medical Center medical ethics committee.

### Clinical examination and imaging

The joints we studied were the distal and proximal interphalangeal joints of the right hand (8 joints per patient). During baseline physical examination, pain upon palpation and soft tissue swelling per joint was assessed.

Radiographs were made at baseline and after 2 years and scored in known time order and blinded for clinical and demographic data according to the Kellgren-Lawrence (KL) method (0–4) (WD) [17]. Intra-observer reliability was high [6]. Radiographic progression was defined as a score increase ≥ 1.

**Fig. 1:**
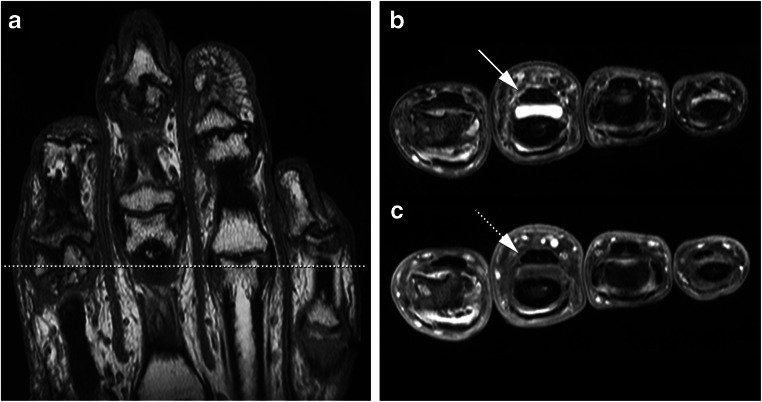
**a** Coronal T1-weighted image without contrast enhancement of the right hand, indicating the location of the axial section (**b** and **c**). **b** and **c** Axial non-contrast-enhanced T2-weighed (T2) (**b**) and axial Gd-chelate contrast-enhanced T1-weighted (T1-ce) (**c**) images showing the proximal part of the third proximal interphalangeal joint, with effusion on the T2 image seen as white water signal (**b**, arrow), with only little enhanced synovial thickening seen as contrast enhancement on the T1-ce image (**c**, dashed arrow) which is not enough for a score “1”

MR imaging was performed at baseline, using a GE-MSK-Extreme 1.5-tesla extremity MR scanner (GE, Wisconsin, USA). Synovitis and effusion give high signal intensity on T2-weighted MR images (T2-hsi) and are therefore indistinguishable. The use of contrast agent enhancement enables distinction and therewith separate assessment of synovitis and effusion. The “synovitis” that is usually scored on Gd-chelate contrast-enhanced MR images [13] is actually thickened synovial lining with increased vascularization. Therefore, we used the term “enhanced synovial thickening” (EST) instead of “synovitis.” Images were scored for four features: T2-hsi, EST, effusion, and BMLs. Details about the MR imaging and scoring protocol were described earlier [6]. Because T2-hsi and effusion are not included in MR imaging scoring systems [13, 14], definitions were made in close collaboration with experienced musculoskeletal radiologists (JB, MR). The scoring protocol for effusion is presented in Table 1, example in Fig. 1.

**Table 1:** Scoring method for effusion

T2-hsi: scored 0–3 using axial non-contrast-enhanced T2-weighted images.	
EST: scored 0–3, using axial Gd-chelate contrast-enhanced T1-weighted images, following the synovitis score of the Oslo hand OA score [13]	
Both T2-hsi and EST scores were defined as follows:	
0 = no synovial high signal or enhanced thickening;	
1 = mild, 1/3 of the synovium with high signal or enhanced thickening;	
2 = moderate, 2/3 with high signal or enhanced thickening;	
3 = severe, all synovium with high signal or enhanced thickening.	
At the time of scoring, T2-hsi and EST were compared with define effusion, which was present in two situations:	
1) T2-hsi > 0 and EST = 0, or	
2) T2-hsi = EST but T2-hsi was scored in a different location than EST in the same joint.	
For both situations, this means that effusion is seen as high water signal on T2-weighted images on a location without high signal on Gd-chelate contrast-enhanced T1-weighted images (for example, Fig. 1).

MR images were scored after training by a radiologist (MR) by two readers (WD, RL), who were blinded for demographic and clinical data and for each other’s scores. Intrareader reliability was good (ICC ≥ 0.9 for all features). For disagreement between readers, consensus scoring was performed.

### Statistical analysis

Associations between the presence of EST or the presence of effusion at baseline and pain and radiographic progression on the joint level were studied using generalized estimating equations, adjusting for BMLs and patient effect. Results were presented as odds ratios (ORs) with 95% confidence intervals (CIs). With the same model, combined effects were evaluated, estimating ORs for pain or progression for joints without EST and effusion as reference versus (1) joints with EST and effusion; (2) with EST without effusion; (3) without EST with effusion. Data for EST and BML were dichotomized into presence (score > 0) or absence (score = 0). Joints with KL score 4 at baseline were excluded from the progression analysis, as they could not further progress within this scale. In the pain analysis, only data of patients who had 3 weeks or less between clinical examination and MR imaging were used. SPSS software for Windows V.20.0/23.0 (IBM, NY, USA) was used.

## Results

### Study population and the presence of clinical and imaging features

This analysis concerned 87 patients (mean age 59 years, 82% women, 89% at least one hand joint with KL ≥ 2). Details of the study group were presented earlier [6]. Data for pain upon palpation were available in 87% (76/87) of patients; the pain was present in 105/608 (17%) joints in 39 patients. Radiographic progression was seen in 78/696 (11%) joints in 45 patients. Soft swelling was present in 49/696 (7%) joints in 21 patients.

In 120/691 (17%) joints in 52 patients, effusion was present, while in 305/690 (44%) joints in 74 patients, EST was present. Remarkably, 57 joints (in 32 patients) showed effusion without EST (57/120, 47.5%). Synovitis and effusion were present together in 63 joints (in 35 patients) (63/120, 52.5%). Soft swelling was present together with effusion in 15/48 (31%) and with EST in 30/48 (63%) joints.

### Association of effusion and enhanced synovial thickening with pain and progression

The presence of effusion was not associated with pain or progression in the same joint: OR 0.8 (95% CI 0.5–1.4) and 0.5 (0.2–1.1), but these ORs showed a tendency towards a protective effect. The presence of EST was associated with pain and progression in the same joint: OR 1.8 (1.2–2.8) and 3.2 (1.9–5.4).

### Combined effect of effusion and enhanced synovial thickening

As the next step, we investigated the effect of the presence of effusion on the association between EST and pain or progression. EST without and with effusion was associated with pain in the same joint (Table 2). EST without effusion was clearly associated with radiographic progression, while this association was only marginal in the presence of effusion. Effusion in the absence of EST showed no association with pain or radiographic progression. Adjustment for BML did not materially change these results.

**Table 2:** Associations for the presence or absence of effusion and enhanced synovial thickening (EST*) with pain and 2-year radiographic progression in the same joint in 87 patients with hand osteoarthritis

Pain			Progression		
	EST	EST		EST	EST
	Absent	Present		Absent	Present
Effusion**	1 (ref)	1.8 (1.1–3.1)		1 (ref)	3.7 (2.0–6.7)
Absent	*n* = 275	*n* = 221		*n* = 319	*n* = 227
Effusion	0.7 (0.2–2.3)	1.6 (0.8–3.1)		0.8 (0.2–2.7)	1.5 (0.6–4.2)
Present	*n* = 50	*n* = 57		*n* = 56	*n* = 60
Adjusted for the presence of BML					
Pain			Progression		
Effusion	1 (ref)	1.7 (1.0–2.9)		1 (ref)	3.2 (1.7–5.8)
Absent	*n* = 275	*n* = 221		*n* = 319	*n* = 227
Effusion	0.7 (0.2–2.3)	1.6 (0.8–3.0)		0.8 (0.2–2.7)	1.3 (0.5–3.1)
Present	*n* = 50	*n* = 57		*n* = 56	*n* = 60

Results are depicted as odds ratios with 95% CIs. *n*, number of joints; *BML*, bone marrow lesions

*EST defined as high signal scored 0–3 on axial Gd-chelate contrast-enhanced T1-weighted fat-suppressed images

**Effusion defined as follows: (1) T2-hsi^#^ > 0 and EST = 0, or 2) T2-hsi = EST but T2-hsi had a high signal on another location in the same joint. ^#^T2-hsi: combination of EST and effusion seen as high signal, which is scored 0–3 using axial non-contrast-enhanced T2-weighted images

## Discussion

We showed that MRI-defined effusion is present in interphalangeal joints of patients with hand OA, in joints with EST (enhanced synovial thickening), but remarkably also in joints without EST. Effusion on itself was not associated with pain or progression, although a tendency towards a protective effect was seen. Contrary to our hypothesis, the known association between synovitis (here: EST) and progression was attenuated by the presence of effusion.

Different mechanisms could explain our results. First, effusion could decrease mechanical stress, leading in the long term to less radiographic damage. Second, the fluid could contain substances that aid repair or could be anti-inflammatory. Third, the presence and absence of effusion could reflect different stages of the inflammatory disease process.

We assessed MR effusion apart from and in combination with EST. An ultrasound study that assessed effusion in patients with hand OA reported that it was associated with pain and radiographic progression, which is contrary to our results [2, 3]. Further differences are the imaging techniques and the hand joints studied. However, the ultrasound study did not investigate the role of effusion apart from synovitis.

Our study has its limitations. First, EST is not the same as synovitis. To assess synovitis, histology should be used, which is invasive and not feasible. We think that EST is the best available biomarker/proxy to assess synovial inflammation. Second, in small hand joints, synovitis and effusion might be difficult to distinguish. Therefore, we set predefined definitions and scored in a conservative manner. From our experience in this study, and supported by previous studies [2, 3, 12, 15], we feel that a distinction between synovitis and effusion is possible even in small finger joints. Third, we did not assess construct validity by comparing MR effusion with another measure such as ultrasound. However, the validity of MR effusion is supported by an earlier study assessing the construct validity of ultrasound effusion using MR imaging as a reference [15]. Nevertheless, validity and (interreader) reliability should be further confirmed before our definition of MR effusion could be used in hand OA MR scoring methods. Fourth, we did not ascertain effusion during follow-up while it is possible that effusion is a transient phenomenon [3, 18]. However, several studies have shown that baseline inflammatory features such as synovitis and BMLs are of clinical relevance for their association with pain and structural progression [3, 5, 6, 19], regardless of their possible transient nature [20]. We think the same applies to effusion and therefore, our results are relevant.

Up until now, effusion is not a feature in scoring methods for hand OA [13, 14]. Therefore, we made definitions in collaboration with experienced radiologists, and consensus scoring was performed with two readers. Therefore, we think our results are valid. We envision including effusion in the MRI scoring system; our scoring method could serve as a blueprint for this. From an earlier MRI study, we learned that increase/decrease in synovitis was associated with increase/decrease in pain [20]. We think that especially this last finding is interesting because the association of a decrease in synovitis with a decrease in joint pain makes synovitis a treatment target. In the present study, we showed that the presence of effusion has an attenuating effect on the effect of synovitis. Therefore, it would be interesting to investigate whether a change in effusion would be associated with a change in pain or a lower chance of radiographic progression over time and how this is related to the effect of synovitis. If future studies can show such effects, we expect that the inclusion of effusion will increase the responsiveness of the MRI scoring system. In conclusion, we found that effusion can be assessed as a separate feature on MR images and is present in interphalangeal joints apart from synovitis. Our study indicates that effusion seems to be of importance for its attenuating effect on the association between synovitis and radiographic progression. Our results warrant further study into the presence and role of effusion apart from synovitis in hand OA, to further elucidate the role of inflammation, and in particular effusion, in hand OA pathology and its treatment.

## References

[1] Berenbaum F (2013). Osteoarthritis as an inflammatory disease (osteoarthritis is not osteoarthrosis!). Osteoarthr Cartil.

[2] Kortekaas MC, Kwok W-Y, Reijnierse M, Watt I, Huizinga TWJ, Kloppenburg M (2010). Pain in hand osteoarthritis is associated with inflammation: the value of ultrasound. Ann Rheum Dis.

[3] Kortekaas MC, Kwok W-Y, Reijnierse M, Kloppenburg M (2015). Inflammatory ultrasound features show independent associations with progression of structural damage after over 2 years of follow-up in patients with hand osteoarthritis. Ann Rheum Dis.

[4] Haugen IK, Bøyesen P, Slatkowsky-Christensen B, Sesseng S, van der Heijde D, Kvien TK (2012). Associations between MRI-defined synovitis, bone marrow lesions and structural features and measures of pain and physical function in hand osteoarthritis. Ann Rheum Dis.

[5] Liu R, Damman W, Reijnierse M, Bloem JL, Rosendaal FR, Kloppenburg M (2017). Bone marrow lesions on magnetic resonance imaging in hand osteoarthritis are associated with pain and interact with synovitis. Osteoarthr Cartil.

[6] Damman W, Liu R, Bloem JL, Rosendaal FR, Reijnierse M, Kloppenburg M (2017). Bone marrow lesions and synovitis on MRI associate with radiographic progression after 2 years in hand osteoarthritis. Ann Rheum Dis.

[7] Lipson RL, Baldes EJ, Anderson JA, Polley HF (1965). Osmotic pressure gradients and joint effusions. Arthritis Rheum.

[8] Rutherford DJ (2014). Intra-articular pressures and joint mechanics: should we pay attention to effusion in knee osteoarthritis?. Med Hypotheses.

[9] Kloppenburg M, Kwok W-Y (2012). Hand osteoarthritis—a heterogeneous disorder. Nat Rev Rheumatol.

[10] Kortekaas MC, Kwok W-Y, Reijnierse M, Huizinga TWJ, Kloppenburg M (2013). In erosive hand osteoarthritis more inflammatory signs on ultrasound are found than in the rest of hand osteoarthritis. Ann Rheum Dis.

[11] Parnell RW (1951). Knee effusion in young adults as an early sign of degenerative arthritis. Ann Rheum Dis.

[12] Agten CA, Rosskopf AB, Jonczy M, Brunner F, Pfirrmann CWA, Buck FM (2018). Frequency of inflammatory-like MR imaging findings in asymptomatic fingers of healthy volunteers. Skelet Radiol.

[13] Haugen IK, Lillegraven S, Slatkowsky-Christensen B, Haavardsholm EA, Sesseng S, Kvien TK, van der Heijde D, Bøyesen P (2011). Hand osteoarthritis and MRI: development and first validation step of the proposed Oslo Hand Osteoarthritis MRI score. Ann Rheum Dis.

[14] Haugen IK, Østergaard M, Eshed I, McQueen FM, Bird P, Gandjbakhch F (2014). Iterative development and reliability of the OMERACT hand osteoarthritis MRI scoring system. J Rheumatol.

[15] Wittoek R, Jans L, Lambrecht V, Carron P, Verstraete K, Verbruggen G (2011). Reliability and construct validity of ultrasonography of soft tissue and destructive changes in erosive osteoarthritis of the interphalangeal finger joints: a comparison with MRI. Ann Rheum Dis.

[16] Damman W, Liu R, Kroon FPB, Reijnierse M, Huizinga TWJ, Rosendaal FR, Kloppenburg M (2017). Do comorbidities play a role in hand osteoarthritis disease burden? Data from the hand osteoarthritis in secondary care cohort. J Rheumatol.

[17] Kellgren JH, Lawrence JS (1957). Radiological assessment of osteo-arthrosis. Ann Rheum Dis.

[18] Kortekaas MC, Kwok W-Y, Reijnierse M, Huizinga TWJ, Kloppenburg M (2014). Follow-up study of inflammatory ultrasound features in hand osteoarthritis over a period of 3 months: variable as well as constant. Osteoarthr Cartil.

[19] Haugen IK, Slatkowsky-Christensen B, Bøyesen P, Sesseng S, van der Heijde D, Kvien TK (2016). MRI findings predict radiographic progression and development of erosions in hand osteoarthritis. Ann Rheum Dis.

[20] van Beest S, Damman W, Liu R, Reijnierse M, Rosendaal FR, Kloppenburg M (2019). In finger osteoarthritis, change in synovitis is associated with change in pain on a joint-level; a longitudinal magnetic resonance imaging study. Osteoarthr Cartil.

